# Putting the Questions First—Flipped Classroom Methods in Animal Ethics Online Teaching and Its Evaluation

**DOI:** 10.3390/ani13050826

**Published:** 2023-02-24

**Authors:** Katharina Dieck, Herwig Grimm

**Affiliations:** Unit of Ethics and Human-Animal-Studies, Messerli Research Institute, University of Veterinary Medicine Vienna, Medical University of Vienna, University of Vienna, 1210 Vienna, Austria

**Keywords:** didactics of ethics, animal ethics, flipped classroom, online teaching, empirical evaluation

## Abstract

**Simple Summary:**

Despite the challenges the pandemic presented for university teaching, it opened up opportunities to set up and explore digital teaching formats like never before. This paper presents a case study in which an introductory animal ethics course was reconstructed as a digital format with flipped-classroom methods. The Interactive Literature Lecturing Format (ILLF) was designed as an online format that maximized interaction with students, without adding further workload to the teaching staff. Rather than provide the students with input in lecture sessions, the ILLF presented students with selected literature and a list of structured questions. This literature questionnaire served as the main didactic element that guided the knowledge transfer, the structure of the sessions and the exam. This paper reviews the outcome of the redesigning process and the steps we took to implement it. To discuss the overall quality of the format from a student’s perspective, the data from the systematically conducted students’ evaluation (*n* = 65) are interpreted using quantitative and qualitative methods. The data suggest that students were overall satisfied with the structure of the course and reported that the flipped-classroom elements worked well. Overall, implementing the ILLF could significantly improve the quality of animal ethics teaching for students and the teaching staff. Therefore, exploring further applications of the ILLF to other fields and on-site teaching should be considered.

**Abstract:**

Despite the challenges the pandemic presented for university teaching, it opened up opportunities to set up and explore digital teaching formats like never before. This paper presents a case study of teaching introductory animal ethics in a digital format with flipped-classroom methods. The Interactive Literature Lecturing Format (ILLF) was designed along the following criteria: 1. Conformity with students’ varying educational needs; 2. Consistent high level of interaction; 3. Maximum transparency in an application-oriented exam; 4. No further contribution to the workload of the teaching staff; 5. Flexibility regarding online or on-site conversions. Rather than provide the students with input in lecture sessions, the ILLF presents students with selected literature and a list of structured questions. This literature questionnaire serves as the main didactic element that guides the knowledge transfer, the structure of the sessions and the exam. This paper reviews the outcome of the redesigning process and the steps we took to implement it. To discuss the overall quality of the format from a student’s perspective, the data from the systematically conducted students’ evaluation (*n* = 65) are interpreted using quantitative and qualitative methods. Bringing these results together with the perspective of the teaching staff, the following question is discussed: did the ILLF meet these criteria? This case study explores the potential and limits of flipped-classroom methods for applied ethics teaching in a university setting.

## 1. Introduction: COVID-19 as a Chance to Rethink Teaching Formats

The pandemic presented numerous disruptions in many areas of life, including university teaching. Right from the start, university teaching around the world underwent severe restrictions, which led to a collective conversion from on-site events to digital formats. Despite the challenges this presented for teachers and students, it opened up opportunities to set up and explore digital teaching formats like never before. This paper presents a case study of an introductory animal ethics course that was significantly improved by reconstructing it in a digital format with flipped-classroom methods.

The course in question, offered at the Institute of Philosophy at the University of Vienna, includes a lecture and reading components. It is taught annually and forms a part of the curriculum for philosophy students in the BA and MA Philosophy programs, as well as for students working through a philosophical extension curriculum. Thus, it is credited in many different modules, but it is not mandatory in any module. It is evaluated with 5 ECTS credit points in the European Credit Transfer and Accumulation System, in which 1 ECTS credit point is equal to 25 working hours. Thus, the course involves 125 working hours in total and is taught in German. As a lecture course, it does not require attendance throughout the semester, as there is only a written exam at the end of the semester for evaluation and three additional exams that take place throughout the following semester. The content is divided into two parts, the first one dealing with traditional theoretical approaches in the animal ethics debate and their critique, i.e., Peter Singer [1], Tom Regan [2] and alternative approaches, such as those of Clare Palmer [3], Cora Diamond [4] and Cheryl Abbate [5]. The second part of the lecture focuses on fields of application of animal ethics, i.e., farm animals, laboratory animals and pets. When they complete the lecture course, students should meet the following three learning objectives:

1. To reproduce central arguments in animal ethics and integrate their corresponding theoretical strands within the current animal ethics debate;
2. To apply the theoretical approaches to practical examples, i.e., to be able to identify ethically relevant aspects in practical situations and interpret them by drawing from animal ethical theories;
3. To critically reflect on animal ethics theories both in their practical application and by comparing them to other arguments in the present debate.

After the first semester was spent merely switching a pre-pandemic lecture format to the virtual classroom, there was room for improvement. Multiple incidents of plagiarism in the final exam challenged the examination format. Transferring the exam from an on-site mode to an open-book, online mode jeopardized any effective control over whether or not the learning objectives were actually met. The exams revealed that it was no longer apparent whether students could reproduce and apply their actual knowledge or whether they were just skilled in sharing and rewriting existing texts. This presented not just an immediate need for change but an opportunity to rethink established teaching methods and structure the course’s contents in a novel set-up, asking anew: which elements define good quality lecture formats, safeguard the learning objectives and can be transferred to an online setting? In answering these questions, the authors identified a list of five criteria that helped guide the design for this new lecturing format. Unfortunately, at the time of the redesign of this format, i.e., in the first months of the pandemic, the transition from ethics courses to online teaching had not yet been adequately addressed in the literature. Therefore, the authors have drawn on their own teaching experience and exchange with students to determine these criteria. The final list of five criteria has been separated into three criteria that qualify a good lecture from the students’ perspective and two criteria that any viable format should meet from a teacher’s perspective.

From a student’s perspective, what makes a lecture format worthwhile is, firstly, that the course should be tailored to the diverse needs of students. As the course in question is an elective and a lecture format does not demand mandatory attendance throughout the semester, the format should enable the students to decide when and how much they invest in the provided program. On the one hand, it should provide possibilities for increased engagement for students who are willing to dedicate more of their time. On the other hand, if students have only limited time resources, the materials provided should enable them to prepare independently and time efficiently for the exam. Secondly, a good lecture format should safeguard a high level of interaction between students and the teacher and allow for in-depth discussions. For those students who attend weekly sessions, there should be sufficient room for questions and discussion points. In order to safeguard this interaction, the format should further offer systematized opportunities for students to report on their experience with the literature so that the teacher can tailor the weekly input according to the students’ needs. Lastly, the exam should be designed in a transparent way that challenges students without causing unnecessary stress. Safeguarding transparency here allows students to prepare for the exam in a target-oriented way and to avoid any guesswork. The exam should position students to demonstrate what they understood, not what they memorized. 

The following two requirements for a good teaching format are from the teacher’s perspective. First, and most important, is that the new format must not permanently increase the teaching workload. This is a precondition to the requirements above, since many ways to meet student needs demand too much work from the teaching staff and are thus unlikely to be implemented. For example, giving individual feedback to voluntary weekly text submissions promises to be very helpful for students. However, correcting more than 20 submissions per week can easily add up to another full working day for teaching staff and, therefore, conflicts with other commitments. The more time-intensive any format is for the teacher, the less sustainable its implementation. Second, a good lecture format should provide sufficient flexibility for the teaching staff. It must be easily adjustable to online or on-site modes of teaching without compromising quality. 

In the following case, the course was designed expecting 40 to 60 students to attend the lectures and participate in the first examination at the end of the semester. Approximately 80 to 100 students were expected for the following three examinations that would take place throughout the next semester, thereby raising the total number of expected students from 120 to 160. The redesign, implementation and evaluation processes were carried out by a team consisting of one professor and an assistant. This paper reviews the outcome of the redesigning process, the steps to implement it and the results of the systematically conducted students’ evaluation (*n* = 65). In the first step, the outcome of the design process, the Interactive Literature Lecturing Format (ILLF) is introduced and its implementation was elaborated on by the teaching staff. In the second step, the materials and methods of the evaluation survey are sketched to interpret the data in the third step. In the fourth step, the perspective of the teaching staff is brought together with the students’ evaluation survey results, representing the students’ perspective. Then the ILLF’s ability to meet the criteria for a good lecturing format is discussed and potential future directions are delineated. A fifth step concludes the case study by explaining the added value of the ILLF as a didactic tool for ethics teaching. 

## 2. Design and Structure: The Interactive Literature Lecturing Format (ILLF)

The course design model that promised to meet all of the requirements is the Interactive Literature Lecturing Format (ILLF). In order to bring the criteria introduced above together in a feasible way, the ILLF draws from the flipped-classroom methodology:

“The flipped classroom teaching model is colloquially defined as one in which the activities traditionally carried out by students outside class (e.g., practicing problem-solving) are moved into the classroom session, whereas what is traditionally completed in class (e.g., expository, information transmission teaching) is completed outside and prior to class.”

(Låg & Sæle [6], p. 1; see also Galindo-Dominguez [7])

For an animal ethics lecture course, flipped-classroom methodology refrains from knowledge transfer consisting of one professor lecturing in front of the whole class throughout most of the lecture. While in a usual lecturing format, the arguments and central concepts of the literature are presented in the lecture by the teacher, in the ILLF, the students gain most of the relevant knowledge on their own by reading the selected literature. The activity that usually takes place outside of the lecture, i.e., developing understanding by putting theory into a practical context, is transferred to the weekly sessions. These sessions are not used to repeat the arguments from the literature, but rather to exclusively apply theoretical knowledge, providing opportunities to ask questions and have in-depth discussions. Therefore, attending the lecture is not beneficial for students who have not read the corresponding texts for the session. The central, flipped-classroom inspired, didactic element is a questionnaire containing a comprehensive list of semi-closed questions that guide readers through each of the texts provided in anticipation of the live discussion (see Appendix A for the full list of guiding questions in the first session of the lecture course). The questionnaire runs through all phases of the lecture course: It facilitates knowledge transfer by systematizing the reading process with guiding questions; The students’ answers to the questions provide the basis for structuring the weekly sessions; The questions cover the exam’s theoretical foundation and can, therefore, systematically guide exam preparation.

The new format begins with an online introductory session that presents an overview of the topics and explains the new format to the students in detail. It includes instructions on how to use the material and the online learning platform (Moodle). Furthermore, it points to interactive tools, such as the discussion forum for students, and a tool that enables students to give feedback anonymously at any time. A detailed breakdown of the credit points into working hours made the requirements for the seminar as transparent as possible. It would take approximately 18 h (12 sessions of 1.5 h) of attendance or listening to the recordings of the sessions, 66 h for reading (11 weekly sessions of 6-h literature preparation), 22 additional hours to fill out the questionnaire (two hours per literature/session) and, lastly, 19 h for final exam preparations. This comes to a total estimated workload of 125 h required for 5 ECTS.

### 2.1. Knowledge Transfer: Guided and Independent Literature Work

For the flipped-classroom methodology, the main challenge from a teacher’s perspective is ensuring the correct understanding of the texts. However, by refraining from lecturing, the teacher also relinquishes control of how the arguments are encountered and thus how students interpret them. To enable a comprehensive understanding of the relevant positions, the teacher must rely on relevant secondary literature, which complements the primary texts. The primary text, e.g., Singer’s *All Animals Are Equal* [1], introduces the original key terms and argumentation styles. The secondary literature for this text, e.g., Der Präferenz-Utlitarimus Singers by Grimm & Wild [8], explains the primary text, positioning it in the wider debate. The questionnaire functions as a third element to safeguard understanding by navigating through the primary reading alongside secondary literature. The questionnaire guides students by focusing on the central concepts and facilitating critical reflection on arguments and positions. For each session, there are five to eight guiding questions, such as, “According to Peter Singer, why are animals to be considered morally?”, or “Explain the following central concepts in Singer’s approach and their significance for his argument: Speciesism and interspecies equality; Universalizability; Vital vs. trivial interests”.

This form of independent knowledge transfer enables students to engage directly with texts according to their own time management. If some students have a higher level of prior knowledge, they can finish reading quickly. This stands in contrast to traditional lecturing, where students would have to sit through a lecture in which the teacher presents arguments they might have already understood. Furthermore, students can give detailed or brief answers as they deem appropriate. If some are not as familiar with philosophical texts, they can repeat central passages and express lingering unclarity through the three questions that complete each list of questions for any session, i.e., “Did the theoretical approach convince you? Why/Why not?”, “Are there any aspects that are not yet clear to you?” and “Are there aspects of Singer’s theory that you would like to discuss in the session?”. These are key for the second function of the questionnaire—structuring the weekly sessions.

### 2.2. Weekly Sessions: Prepared In-Depth Discussions

The answers to the literature questions could be uploaded to Moodle for the teacher to analyze the submissions before the weekly session, three hours at the latest before the session. This submission deadline provides a fixed timeslot that is sufficient for the teacher to adjust the sessions based on student input. The uploaded answers (on average, 17 submissions per week) provided the exclusive basis for structuring each session. This allows the teacher to prepare the sessions time efficiently and make them target-oriented, as there is no need for slides that introduce arguments in the first place. As the lecture format allows for non- or irregular attendance, any submissions throughout the semester were on a voluntary basis. Thus, submitting the answers to the questionnaire via Moodle is an offer from the teacher to the students to partake in designing the content of the weekly sessions. The weekly discussions take place via Zoom only and are recorded and uploaded to Moodle for students to rewatch. Within the weekly discussions, no exam-relevant material is introduced. However, in taking part in the discussions regularly, students also deepen their skills to apply theories to practical situations and to reflect on ethical theories critically, which is vital for the exam (see learning objective 2 and 3).

The discussion is broadly structured into two parts. The first one focuses on resolving any confusion from the students’ reading. When reading the students’ submission to the questionnaire, the teacher checks whether there are recurring misunderstandings from the reading, e.g., connecting the idea of animals having dignity to Singer’s approach. If there are, the teacher quotes them in slides for the presentation and explains what the misunderstanding consisted of. Furthermore, what students found unclear in their submissions is directly addressed, ensuring an overall understanding of the theoretical positions, e.g., “Does the interests of a being have to be consciously experienced in order to count morally?”

The second part of the discussion consists of deepening and applying the theoretical knowledge. The students’ comments regarding what they would like to discuss are scanned by the teacher. The most common comments from the submissions are addressed in the session, e.g., which beings we can safely ascribe sentience to and questioning the idea of self-conscious beings and non-self-conscious beings. In some sessions, it is possible to present real examples highlighting particular challenges for theoretical approaches. In the third session, Clare Palmer’s approach to the distinction of wild and domesticated animals [3] is discussed using the example of rewilding in a nature reservoir in the Netherlands, Oostvaardersplassen. As a rewilding project, animals are left to themselves, which leads to animal suffering due to limited nutritional resources and overpopulation, thereby motivating activist intervention [9]. The reserve, in its function as a nature protection zone, is fenced, which hinders wild animal movement and foraging. Scarce winter resources were especially hard on the overpopulated wild horses. This illustrated a critique of the categorization of wildness based on whether or not animals are located in unimpacted environments. As there are barely any unimpacted zones for animals, according to this distinction alone, animals can barely ever be considered wild.

To guide the discussion, the teacher encouraged participants to raise their hands and pose contributions via the microphone, as well as via chat. The chat kept the discussion going by allowing the teacher to pose a question and ask for quick and short answers in the chat. From the chat conversation, the teacher selected interesting short answers, asking students to elaborate on them. Furthermore, this enabled students to point out unclarities right away, which was helpful for the teacher as well, provided that there was sufficient time to filter through the questions in the chat.

Overall, it was not the teacher who gave the input in the lectures, but the students themselves. Uploading the answers to the questionnaire regularly invited students to partake in the design and structure of the sessions. The teacher just selected and structured the input. This guaranteed a maximum level of interaction with the students, while maintaining a comparable level of working hours for the teaching staff compared to more traditional lecturing formats. 

This has been reflected in the quality of the discussions in the sessions via Zoom that the teaching staff experienced. Everyone attending was prepared for the discussions by having read the texts and students were generally very engaged. Since the lecturing did not entail content transfer, there was sufficient time to broaden the debate around animal ethics so that each session added further literature to the platform for interested students. For the teacher, the discussions were also more engaging due to the participants’ knowledge. All of this resulted in a significant increase in the teacher’s satisfaction with preparing and moderating the weekly sessions, even though the total amount of working hours put into the preparation did not decrease but remained roughly the same. However, the hours invested into preparation via the students’ submissions were more purposeful due to the immediate interactions with the explicit students’ demands in understanding the texts.

### 2.3. Exam: Transparent and Beyond Knowledge Reproduction 

The third and final function of the questionnaire was especially important for meeting the requirement to tailor the lecture to the diverse students’ needs. It provided the theoretical foundation for the exam, i.e., no exam questions went beyond what was addressed by the questionnaire. If the questionnaire was filled out properly, students would not face topics beyond their knowledge in the exam. The questionnaire thus served as a guide not only through the literature, but also through any independent exam preparation. Since any lecture has to provide four exams in total, with no required attendance during the semester, it was common for students to study exclusively on the basis of the materials provided and prepare independently for the exam. The questionnaire facilitated this way of studying, as it allowed for consecutive preparation. Since it contained the condensed contents of the lecture, it was helpful in refreshing knowledge. Students could, for example, start at the beginning of the semester with active engagement in the course, pause it and then pick up studying at the end of the semester again without having wasted any time. Alternatively, students who regularly attend the sessions and submit answers to the weekly questionnaire may have to study less. The more hours one puts in during the semester, the less one has to prepare for the exam. 

To keep such a balance, the exam is designed in two parts, testing whether the learning objectives were met. The first part is the independently filled-out questionnaire that covers the first learning objective, i.e., to reproduce central arguments in the animal ethics debate. If the questionnaire is filled out properly, the reproductive knowledge requirement is fulfilled, because uploading a filled-out questionnaire on the exam date is a prerequisite to participate in the exam. The questionnaire is not evaluated in terms of quality, since this would increase the workload involving the evaluation drastically. Plagiarism software tests reveal whether or not the submissions were filled out individually. This is essential insofar as it prevents students from circulating filled-out questionnaires and using them as the sole material for preparation and re-uploading answers on the exam. As helpful as the questionnaire is, without this precaution, the ILLF is vulnerable insofar as it allows students to surpass any original texts in the exam preparation. The mandatory uploading of the filled-out questionnaire before the exam also reduces the number of participants who go into the exam heavily unprepared and thereby decreases the workload of evaluation for the teacher afterwards. 

The second part is an application-oriented, open-book, 2-h take-home exam. This part covers the second and third learning objectives, i.e., applying theories to practical examples and critically reflecting on them. For the second part of the exam, the students receive two relevant case scenarios in animal ethics to choose from, together with the exam questions via e-mail. They have to upload their answers to Moodle within two hours. As the reproductive basis has been covered by the first part of the exam, they are welcome to use any material for addressing the questions. Due to this division of the exam format, the aim of the take-home exam is for students to show that they understand the theoretical approaches and can thus apply them and reflect critically. 

From a student’s perspective, the online format has the benefit of allowing to participate in the exam from any place. The two-hour limit minimizes the potential length of the answers, as it is expected that answers tend to become longer the more time is provided, which would increase the total evaluation hours of the teacher. Furthermore, two hours are sufficient for students to demonstrate an understanding of the lecture contents. The exam is designed in an application-oriented format, i.e., students have to analyze the animal ethics scenario with the theoretical approaches learned in the lecture. The scenarios are roughly a quarter of a page long and display challenging dilemmatic situations for a person who has to make a decision. For example:

“Martina keeps five hens and a rooster in her backyard in a coop with an outdoor area. She and her family enjoy the fresh eggs the hens lay every day. One day she notices that one hen has started to hatch three eggs. However, the coop is clearly too small for a total of nine hens, and their quality of life would suffer from the limited freedom of movement. What should Martina do?”

The exam questions then provide the interpretative frame for analyzing the case examples, for example:

“Elaborate on the morally relevant aspects in this example against the background of the theories by Peter Singer and Tom Regan. In doing so, discuss possible answers to the question “What should Martina do?” and explain the central differences between the two approaches by illustrating them with features of the example.”

Overall, theoretical approaches are utilized to interpret the scenario and reflect on the theories, e.g., where do problems arise in their application? Thus, the online take-home exam provides the opportunity for students to apply and further explore their knowledge on a case-by-case basis rather than reproducing memorized knowledge. 

### 2.4. Evaluation of the Exam

In order to evaluate the exams, teaching staff checks whether both documents, i.e., the filled-out questionnaire and the answers to the exam questions, were submitted. Otherwise, the exam cannot be passed. The exams are then pre-evaluated by assisting staff before the teacher grades them. The pre-evaluation follows a template that is constructed for each exam individually. It breaks down the maximum points for each question into requirements for the answer. A good answer to the question above regarding Singer’s approach should, for example, mention the concepts of speciesism, interspecies equality, universalizability and vital vs. trivial interests, illustrating it in the example provided. Furthermore, the pre-evaluation looks at how the question is answered: Is it correct? Did the participant apply the theories to the example sufficiently? Is sufficient justification provided? Lastly, the style is evaluated, i.e., is the answer clear and well structured? Pre-evaluating exams in this structure reduces the time invested by the teacher in grading to a minimum. 

Switching the examination method to an open-book and application-oriented format increases the satisfaction of pre-evaluating and grading the submissions significantly for the teaching staff. This is because the answer quality is higher, and it is thus more interesting to read the answers. Furthermore, this format allows for more diverse answers and approaches from students, which makes evaluating also more diverse. This offers students flexibility when taking the exam and the teaching staff when evaluating answers, as two very different approaches to one and the same question can be equally right. With these application-oriented, reflective questions, it is not about what the student determines to be the answer but how they argue for it.

## 3. Evaluation Survey: Materials and Method

In order to gather a comprehensive view of the students’ perspective on the ILLF, an evaluation form was sent out to the students after each examination date. The exam is the element which eventually reveals how the flipped-classroom knowledge transfer worked for students overall, including those students who never participated in the weekly sessions. Thus, it is crucial to evaluate the students’ experiences not just throughout the semester but on the exam as well. That is why the evaluation targeted those students who were registered for an examination date after the corresponding exam. To gain targeted insights into the students’ perspective on the ILLF and its elements, evaluation forms were specifically designed via the established online survey tool, *Umfrageonline.com*. Using this tool, the links to the evaluation forms were generated and the responses were collected.

### 3.1. Study Design and Measurements

The links to the evaluation were sent by e-mail within 24 h after each examination date exclusively to the students who were registered for the respective examination. This was carried out using the platform of the central teaching organization platform of the University of Vienna (u:space), which allows e-mails to be sent to the registered student accounts as blind copies. After each of the four examination dates, two links were sent out, which enabled the evaluation of two separate groups of students with different evaluation forms. The *active* group consisted of those students who regularly participated in the lecture during the semester. The *non-active* group consisted of those students who independently prepared for the examination with the documents provided. The students were informed that participation in the evaluation is anonymous, on a voluntary basis and has no effect on the evaluation of the exams. The online survey was open for 30 days after sending out the link. After 30 days, the answers were downloaded as Microsoft Excel documents for data analysis. Despite sending out the evaluation forms after the examination dates, the response rate was sufficiently high to allow for conclusions on the quality of the format based on students’ perspectives. A total of 132 students participated over the course of four examination dates, out of which 65 filled out the evaluation forms, resulting in a response rate of 49%.

The evaluation after the examination with independently designed evaluation forms enabled a comprehensive evaluation tailored to the structure of the format. The evaluation forms included both closed and open questions. If not indicated otherwise, only single responses were possible. Closed questions with a numeric scale were integrated to establish comparability between individual assessments for those aspects that were most relevant for future targeted modifications of the format for both groups. This included the estimated amount of work compared to other lectures and the effort invested in exam preparation. Students could evaluate this from 1 to 5, 1 being too low and 5 being too high. The difficulty of the exam could be evaluated from 1 to 5, with 1 being too easy and 5 being too difficult. The amount of information about the format and the exam could be evaluated on a scale from 1 to 5, 1 being too little and 5 being too much. The overall format could be evaluated from 1 to 5, 1 being very good and 5 being very bad. Furthermore, the evaluation form for the active group asked students to assess the quality of the discussions in the sessions from 1 to 5, 1 being very good and 5 being very bad.

The evaluation forms were supplemented by further closed questions with several answer options to understand how students used the flipped-classroom tool. Students from the active group were asked how regularly they prepared the literature and answered the guiding questions, choosing between six answers: prepared literature and guiding questions each week; prepared literature and guiding questions almost each week; prepared literature and/or guiding questions sometimes; prepared literature sometimes but never prepared guiding questions; prepared literature and/or guiding questions rarely; and never. Both groups were asked whether the literature questionnaire was perceived as helpful on the following scale, which allowed for multiple responses: yes, to work with the literature; yes, to systematically prepare for the exam; not really; no; and other (including the possibility to state a short open response). Students from the non-active group were further asked whether they felt sufficiently prepared by working through the material independently on the following scale: yes, very much; yes, sufficiently; no, too little; and no, not at all. 

Open-ended questions captured more closely the motivation of the students to register for the lecture course in the first place. Both groups were asked why they decided to register for this lecture. The non-active group was further asked why they chose not to attend the sessions regularly during the semester. These dimensions, however, were included only to define the target group more precisely for future semesters and do not reflect the quality of the ILLF. They are, therefore, not of further interest here. The final open question posed to both groups was intended to give students the opportunity to evaluate the lecture in their own words, i.e., what they particularly liked about the teaching format and where they see potential for improvement. 

### 3.2. Quantitative Data Analysis

For the quantitative analysis of the responses in the numeric and Likert scales, the data have been systematized with Microsoft Excel, and the overall responses of all evaluations have been divided into one document per group (active vs. non-active). Furthermore, one document has been created containing all the responses in total. The relevant mean values, their standard deviations, and percentages have been retrieved for each group using statistic operators provided by Excel.

### 3.3. Qualitative Data Analysis

The responses to the open questions in the evaluation form (*n* = 53) have undergone a qualitative content analysis that systematizes the raw material into categories to reflect on and stress regularly mentioned points, (see the methodology of Mayring [10]). The first step consisted of scanning the text corpus and noting relevant points. The second step involved clustering the most frequently recurring relevant points into categories. These categories then served in a second analysis of the material. Thus, the categories were retrieved directly from the material, even though the exclusion of points targeted by the closed question could be considered a form of negative pre-selection of categories for this analysis. Consequently, the material was scanned again according to the inductively developed category system and coded according to correspondent keywords of the categories, e.g., sentences containing the keywords or phrases “questionnaire,” “literature guiding questions” or “questions for literature” were coded as the category “questionnaire.” Due to the size of this corpus, there was no need to develop further subcategories. 

## 4. Results

### 4.1. Quantitative Results

Of the overall participating students (*n *= 65), 21 (32.3%) reported to have been active throughout the semester, 19 (90.5%) of which registered for the first examination date. Thus, 44 (67.7%) students claimed to have studied for the exam independently from the lecture sessions. The data suggest that students were largely in favor of the ILLF, as the mean value of the assessment is 1.37 (±0.63) on a scale from 1 to 5, 1 equaling “very good” (see Table 1). In fact, 20 (95.2%) of the active students and 42 (93.3%) of the non-active estimated the format to be at 1 or 2. This shows that the ILLF worked for both targeted student groups alike, with a slightly higher tendency of the non-active students to consider it very good.

How the overall satisfaction is composed was inquired by questions focusing on the central didactic elements in different phases of the semester. The questionnaire was regarded as helpful for guiding through the literature by 12 (57.1%) of the active students and by 31 (68.9%) of the non-active students. Furthermore, it was reported to systematically help prepare for the exam by 17 (81.0%) of the active students and 38 (84.4%) of the non-active students. Only 1 (1.54%) person in total evaluated it to be not particularly helpful. This suggests that the students overall reported the questionnaire to work well as a didactic tool, but more so in terms of structuring the exam preparation than guiding through the literature.

In addition to this, the active group flexibly adopted the questionnaire, as the answers regarding how regularly they prepared the literature for the sessions and fill out the questionnaire varied: fourteen persons (66.6%) stated that they prepared for the session every week or almost every week, while four (19.1%) sometimes prepared the literature or answered the questionnaire and three (14.3%) rarely or never prepared literature or filled out the questionnaire in advance of the weekly sessions.

The active students evaluated the discussions on a scale from 1 to 5, 1 being “very good” and 5 being “very bad”, as on average 1.71 (±0.90). The workload, compared to other lecture formats, was generally estimated higher. On a scale from 1 to 5, 1 being too low and 5 being too high, the active group rated it at 3.76 (±0.70) and the non-active group on 3.64 (±0.66). However, only 6 (11.1%) students overall estimated the workload as generally too high and no one estimated it to be too low.

The final phase of the lecture, i.e., the exam, was evaluated using three questions. Both groups estimated their exam preparation on a scale from 1 to 5, 1 being “very easy” and 5 being “very hard” as 2.40 (±0.86) in total (active group 2.41 (±0.74) and non-active group 2.41 (±0.92)). Furthermore, 41 (93.2%) of the non-active participants claimed to have felt “very prepared” or “sufficiently prepared” for the exam by studying independently with the materials provided. Both groups evaluated the exam as adequately difficult on a scale from 1 to 5, 1 being “very easy” and 5 being “very difficult”; the active group rated it as 2.62 (±0.67) and the non-active group as 2.64 (±0.65). None of the participants found it very easy or very difficult, suggesting that the overall exam format worked for the students as well.

### 4.2. Qualitative Results

The overall impression that the ILLF was received well by the students is solidified through the comments to the open question, which asks what they particularly liked about the seminar and where they see potential for improvement. Recurring issues pointed out by students fall into six categories: format, discussion, interaction with teaching staff, selection of literature, workload and exam. The following section will focus on points brought forward by the students regarding these categories, which go beyond what has been targeted by the quantitative results. 

#### 4.2.1. Format

The impression from the quantitative evaluation that the format was received well by the students could be bolstered by analyzing the written comments. Students described the format as “great” and “very successful,” especially pointing out that the questionnaire was “genius” or “incredibly good.” Furthermore, they stated that “the flipped-classroom concept was implemented well” and that even “away from covid restriction […] this format [is] fantastic.” Participants from the non-active group also commented that they were “not feeling at a disadvantage compared to others who attended the lecture on a regular basis”. 

#### 4.2.2. Discussion and Interaction with Teaching Staff

The evaluation of the discussions, however, is more diverse. On the one hand, students pointed out how “interesting” and “extremely engaging” they found the discussions to be, that “time flew by” and, overall, the discussions were of “added value” for the lecture. Students mentioned that the structure of the sessions was flexible and that the teacher was competent in moderating and embedding single contributions into the larger animal ethics debate. Also, students from the non-active group reported rewatching the recorded discussions to deepen their knowledge, even stating that they regretted not having been able to participate in the discussions. On the other hand, students reported that the discussions were sometimes far off-topic from the literature. They felt that “the red thread was missing” and that the structure of the discussion was too “associative and free” or “excessive” in areas that were not relevant for exam preparation. Therefore, a few students reported missing reassurance as to whether they understood the central arguments correctly. They wished discussions were restricted to the sessions’ literature and guiding questions. 

Furthermore, students pointed out that the interaction with the teacher was “pleasant,” “motivating above average,” “respectful” and “at eye level”. Participants from the non-active group also said they appreciated the “maximum transparency” in the communication about the structure of the lecture, and especially the exam. 

#### 4.2.3. Selection of Literature and Workload 

Students reported that they found the selection of literature to be “good” and “interesting,” but regularly pointed out that particularly texts in the English language added significantly to the workload and that they would have liked more translations. Another issue brought up regarding the literature directly connects to the next category; that the literature selection was too comprehensive and thus created a higher workload than other lecture formats. Furthermore, they reported that filling out the questionnaire was time-consuming, albeit students claimed that it turned out to be worth the effort, as it reduced the time for exam preparation drastically. Still, the overall impression remained that the workload was higher compared to other lectures. 

#### 4.2.4. Exam

The format of the exam, however, was generally appreciated for its being “very pleasant,” “very good because it was application-oriented” and the exam questions being “very exciting, because you had to deal with the material in a different way.” The separation of the exam into a reproductive part (filled-out questionnaire) and the application part (take-home exam) was evaluated positively, as students did not need any further exam preparation when the questionnaire was filled out properly. 

### 4.3. Limitations of the Survey

The time for the evaluation survey was set 24 h after students took the exam, to have students evaluate the exam experience as well. Sending the evaluation forms this late, however, came at a price: Only those students who took the exam were targeted for the evaluation. We did not manage to include those students who refused to take the exam altogether, e.g., because the readings were too comprehensive, or they did not like the format in the first place. As the number of deregistration, which has been retrieved from the online administration system of the University of Vienna (u:space), is rather high compared to other semesters when the same lecture was offered in its more traditional format, the overall drop-out rate of this course seems to be relatively high. In the winter semesters of 2019 and 2020, 29% and 25% of the once-registered students for the four exam times deregistered again. In the winter semester of 2021, when the ILLF was adopted, the deregistration rate was 44%. Thus, 44% of the students who possibly intended to take the exam for the lecture eventually decided against taking the exam. It is worth noting here that there are no numbers available for when students deregistered after having registered; it is unclear whether it was shortly after registering, when reviewing the material and the requirements or when preparing for the exam had already started.

Even though it would have been interesting to examine the reasons for drop-outs, this particular group of students was difficult to target. The interaction with them probably only consists of them looking at the online learning platform, the selection of literature and the requirements. If there were particular reasons for dropping out throughout the semester connected to the format, the anonymous feedback tool provided via Moodle could have been used. The question thus arises, even if one could target this group of students, on what grounds would their evaluation be based?

Sending out an evaluation form at this late stage of the lecture course also gives rise to students’ biases. Firstly, students who are outstandingly happy with the format are more likely to report this than those who found it merely average. Similarly, students who are very dissatisfied with the format or the exam are expected to show more initiative when it comes to evaluating. Since these biases tend to level with each other in the final findings, the results can still be regarded as reliable.

Furthermore, it must be admitted here that the evaluation data from this semester alone only allow for a limited interpretation of the results. To make definite statements on the success of the redesign with the ILLF, a systematic comparison with prior semesters would have been necessary. As the redesign initially prompted the intensive student evaluation, there is no comprehensive set of evaluation data for prior semesters to allow for systematic comparability. Considered on its own, the data can only tell how the transformation to the ILLF and online teaching mode was experienced by teachers and students, or which specific elements have worked well and which have not.

## 5. Discussion

### 5.1. Does the ILLF Meet the Established Criteria for a Good Lecture Format?

Designed amidst the restrictions of the pandemic, the ILLF significantly improved the animal ethics teaching beyond that. Both from a student’s perspective as well as from a teacher’s perspective, the implementation of a structured flipped-classroom format resulted in a high quality of interaction and proved to be viable for students with varying studying styles. Therefore, the ILLF met three initially posed requirements for a good lecture format for students, namely that it should be tailored to diverse students’ needs, offer a high level of interaction with the teacher and be transparent in the way the exam is set up. Firstly, the ILLF effectively managed to target the two central student groups equally. Students who regularly attended the lecture throughout the semester reported being satisfied with the format as well as students who only prepared for the exam individually. It thus allows for maximum flexibility in the students’ time management, which is crucial, especially for those students with work or other commitments. Furthermore, the possibility to submit answers to the literature guiding questions (i.e., questionnaire) each week enabled the teacher to interact with the students and prepare the sessions according to their interests. Therefore, each session is designed by the input of the students and not by the teacher’s interests. This provided maximum room for application-oriented discussions that enabled students to grasp the bigger picture within the debate. Lastly, the two-part exam afforded students a level of transparency, as one part was the direct outcome of the exam preparation, i.e., the filled-out questionnaire. Moreover, the format of the exam provided another way for students to demonstrate their knowledge without the stress of memorizing since, due to its application orientation, it was an open-book examination. 

From a teacher’s perspective, the requirements of a good lecture format could be met overall as well. The format allowed for sufficient flexibility in times when the teaching mode was subjected to quick changes. The ILLF was implemented as an online teaching format and it worked well as such. Nevertheless, it is possible to make the same adjustments to weekly on-site sessions. This would require only a few alterations, but it is yet unclear whether the quality of discussion remains equally high in an on-site mode, for central elements of structuring the discussion are missing, e.g., the teacher cannot use the chat easily to engage students in the discussion by asking for short answers. Furthermore, it can be expected that those students on the quiet side may be more hesitant to participate in discussions in a full classroom of more than forty persons. The format of the exam should also remain online, as it is an open-book format. This is because of the plagiarism software that is required to check the students’ submissions for authenticity: hand-written exams cannot be easily uploaded for plagiarism checks. Even if students were on-site during the exam and later submit their answers digitally, there would be no reason for them to come on-site in the first place. Therefore, the exam structure only makes sense as an online format. If one wanted to provide open-book formats for students, there is no good reason to ask them to come on-site for the exam. On the other hand, a more immediate interaction between students and teachers in future semesters might support the learning objectives of this course. In this case, their skills regarding the application of theories and their critical reflection might benefit from an on-site transfer. 

The second requirement for a good lecture format from a teacher’s perspective, i.e., keeping the workload minimal, was met. The time invested by the teacher throughout the semester in designing each lecture remained largely the same, even though it was much more motivating to react directly to the student’s educational needs. Still, the effective time-saving will only become apparent in future semesters. Implementing the ILLF constituted a great deal of work: Selecting the literature, creating the questionnaire, setting up all relevant documents and transferring the information to the learning platform. However, this format is promising for future semesters—all it takes is a few clicks in the online learning platform to reinstall it for another semester. This is only possible because of the high level of interaction with the students, where each session is tailored according to student input. This level of interaction that is integrated into the framework makes it easy to react to current trends in the debate without having to alter the program at large. When it comes to the overall time invested by teaching personnel in correcting the exams, it is roughly the same as traditional lecturing formats. In order to receive the take-home exam, the students must have filled out the questionnaire beforehand and therefore have a solid knowledge basis to succeed in the exam. Making the upload the questionnaire a prerequisite part of the exam effectively reduces the number of those students who come underprepared. Nevertheless, it must be said that the experience of the teaching staff only allows for limited conclusions as the number of teaching staff involved here was limited (*n* = 2). 

### 5.2. What Can Be Improved in the ILLF? Learning from Students’ Input

Overall, the results of the regular student evaluations showed that the ILLF did not only meet the predefined requirements, but revealed that not only the satisfaction of the teaching personnel was high. However, the evaluations can also be a tool to reflect on the format further. Two issues stand out from the written comment section of the evaluation, i.e., the discussion and the workload. The comments suggest that, for future semesters, it might be worthwhile to orient the weekly sessions more toward the students’ input to provide more guidance when it comes to exam topics. The ILLF challenges students to work independently with literature, which allows for a flexible framework. This may be harder for some students. The question that arises here is whether one should tailor discussions to those students who tend to feel more lost because of the literature and thereby risk other students being underchallenged. Overall, it is advisable to tailor the discussion along the submitted answers to the guiding questions while allowing for extensive discussions for other students. Any insecurities regarding the understanding of the topics might be better dealt with outside of the weekly lectures, e.g., in student reading groups or regular tutorials with a student assistant. 

Regarding the workload, it makes sense to accommodate the students’ wishes to a certain degree and slightly reduce the selection of mandatory readings. The question that arises here is what to align the expected workload with. Should one align the workload with what students expect to do in the framework of their university? Or should one use the credit points (ECTS) as a baseline to decide upon an expected workload? For future semesters, the authors will continue to use the ECTS as a measure to guide the expected workload, for a good lecture should be challenging to a certain degree. This is crucial, especially in undergraduate courses, where students must build the basic habits for efficient text work. Furthermore, providing current research literature from the animal ethics debate will remain a central part of the lecture. As such, it will often be in English, which introduces the debate’s key terminology to the students. 

### 5.3. Future Directions

Redesigning the animal ethics lecture format was illuminating in many ways. What stands out most, however, is not directly connected with the quality of teaching. Evaluating the teaching format so regularly revealed an insight that can redefine what a lecture is about—two-thirds of students that take the exam study independently. Thus, when giving a lecture, two-thirds of the teacher’s target audience is not in the room. The implementation of ILLF and all the material provided is, therefore, why so many students take the exam independently from weekly sessions. This clearly shows, however, that there was an interest in such formats among students. It is an interest also recognized in most curricula offering both mandatory attendance, such as seminars, and those without the need for attendance, such as lecture courses. One could argue further that, for lectures to conform to the format of non-mandatory attendance, the teaching staff has to provide sufficient materials for students to prepare for the exam independently. If a lecture does not provide sufficient material besides the weekly sessions, students are obligated to physically attend to take the exam. The implementation of ILLF has further demonstrated that knowledge transfer does not necessitate attendance. Students are able to learn independently if provided with well-structured material and sufficient room for interaction. Thus, in order to offer student-friendly formats, it is crucial to not only have the independent learners in mind, but to think of them as the majority of the target group and, respectively, realign priorities in lecture formats. This way, teachers can establish fair formats that respect students’ varying time resources and thereby create flexible formats that make participating in worthwhile.

This creates a paradoxical situation, as a third of the students that are regularly present produce the content for the other two-thirds. Even though the majority of students are “absent participants,” one has to make the weekly sessions as attractive as possible for the ones that participate, so as to safeguard good discussions for others to rewatch. Thus, attending weekly sessions should provide added value for students through close interaction between students and teachers. If there is no room for discussion or no further explanations in the weekly sessions, a large part of the material for the non-active group of students is lost. 

Implementing the ILLF sheds a different light on the role of the teacher: the teaching person is no longer the primary knowledge mediator, as students have to extract the knowledge from the literature themselves. Rather, the teacher works as a guide or a facilitator for students to retrieve the information from selected literature. Even though this change of role promises to impact the learning outcomes of students more immediately, it imposes some limitations on potential applications of the format. Firstly, the teacher has to have sufficient expertise on a topic to have the competence to apply it in a flexible setting. The teaching staff must be able to embed students’ criticism and questions regarding their theories into a larger theoretical framework regarding the animal ethics debate. Secondly, the teacher must be comfortable in that particular role and willing to take the risk of going along with the students’ input. This includes preparing slides not to provide more information, but rather to entertain questions and discussions that may go beyond the provided literature. Lastly, the teacher must have good moderating skills to react to students and embed their input into a larger debate. Overall, this particular role might not suit everyone, but, as this case study has shown, there are good reasons to try becoming accustomed to flipping the classroom. 

Taking all of this into account, we regard any further application of the ILLF, be it in an online or on-site format, as desirable. This is especially true because transferring to an on-site mode promises to be insightful for future semesters. Only then can it be discerned whether ILLF actually works well as an online format or whether it has worked well despite being an online format. It is possible that the ILLF can be further improved upon in an on-site mode with all the benefits of more immediate interaction. 

## 6. Conclusions

This case study presented the implementation of an online teaching format that was developed by the authors as a reaction to the pandemic restrictions on university teaching. Taking this setting as the opportunity to explore digital formats, the authors drew from flipped-classroom methodology to design a novel lecture format, the Interactive Literature Lecturing Format (ILLF). The ILLF has been presented here as a structured didactic tool that introduces a novel format to recurring lecture courses with reasonable effort. It not only displays a high quality of the students’ engagement with animal ethics due to the independent knowledge transfer and the consequently engaging discussions, but it is also viable and enjoyable for the teaching staff. The evaluation survey further shows that the students’ satisfaction was very high throughout all phases of the lecture course, regardless of whether they were actively participating or whether they prepared independently for the exam. The ILLF restructures the lecture in a way that enables the teaching staff to reimplement it each semester without any alterations or compromises in the quality of teaching, as the high level of interaction with students is embedded within the structure of the ILLF. Thus, it is promising not only to remain with the ILLF for the animal ethics lecture discussed here, but to explore its further potential in other teaching areas, in other fields and in on-site teaching. However, when it comes to online-teaching, this case study has shown that it should not be regarded as a deficient or second-choice option per se. The implementation of the ILLF in an online mode shows that there are lecturing formats that can ensure high-quality teaching online. Thus, online teaching should be regarded independently from on-site teaching, as a didactic mode in its own right, that can obtain very good outcomes for both students and teachers, if the format actually fits the setting. Therefore, teachers should be encouraged to explore novel digital formats beyond any pandemic restrictions. 

## Figures and Tables

**Table 1:** Students’ evaluation of the Interactive Literature Lecturing Format (ILLF): Comparison between active and non-active group.

		Active (*n* = 21)	Non-Active (*n* = 44)	Total (*n* = 65)
Overall evaluation of the format Mean ± Std		1.48 ± 0.75	1.32 ± 0.56	1.37 ± 0.63
Workload compared to other lecture formats * Mean ± Std		3.76 ± 0.70	3.64 ± 0.66	3.69 ± 0.67
Questionnaire ** Count (%)	Helpful for guiding text work	12 (57.1)	31 (68.9)	43 (65.2)
	Helpful for exam preparation Not particularly helpful	17 (81) 0 (0)	38 (84.4) 1 (2.3)	55 (83.3) 1 (1.5)
Perceived difficulty of exam preparation Mean ± Std		2.38 ± 0.74	2.41 ± 0.92	2.40 ± 0.86
Perceived difficulty of exam Mean ± Std		2.62 ± 0.67	2.64 ± 0.65	2.63 ± 0.65

* Please note that there has been an inconsistency in the numeric scale on the evaluation forms in the two collectors for the first exam date. The numeric scale for the non-active group’s evaluation form ranges from 1 to 7 instead of 1 to 5. Thus, the responses (*n* = 11) to this question in this collector have been excluded. ** Multiple answers were possible.

## Data Availability

Not applicable.

## Appendix A

The first session deals with Peter Singer’s position. Primary Literature: Peter Singer, *All Animals Are Equal* (1976) [1]. Secondary Literature: Herwig Grimm & Markus Wild, Grimm, Der Präferenzutilitarismus Singers (2016) [8], (pp. 57–78). Both texts are mandatory readings.

### Literature Guiding Questions, 1st Session

1. According to Peter Singer, why are animals to be considered morally?
2. Explain the following central concepts in Singer’s approach and their significance for his argument: speciesism and interspecies equality; universalizability; and vital vs. trivial interests.
3. In what way does Singer categorize life hierarchically?
4. What is “the argument from marginal cases”?
5. How does Singer weigh up different interests against each other?
6. Did Peter Singer’s approach convince you? Why/Why not?
7. Are there any aspects that are not yet clear to you?
8. Are there aspects of Singer’s theory that you would like to discuss in the session?
